# Brain age and cognitive functioning in first-episode bipolar disorder

**DOI:** 10.1017/S0033291722002136

**Published:** 2023-08

**Authors:** Trisha Chakrabarty, Sophia Frangou, Ivan J. Torres, Ruiyang Ge, Lakshmi N. Yatham

**Affiliations:** 1 Department of Psychiatry, University of British Columbia, 2255 Wesbrook Mall, Vancouver, BC, Canada V6T 2A1; 2 Department of Psychiatry Icahn School of Medicine at Mount Sinai, New York City, NY, United States; 3 British Columbia Mental Health and Substance Use Services, Vancouver, BC, Canada

**Keywords:** Bipolar disorder, brain-age, cognition

## Abstract

**Background:**

There is significant heterogeneity in cognitive function in patients with bipolar I disorder (BDI); however, there is a dearth of research into biological mechanisms that might underlie cognitive heterogeneity, especially at disease onset. To this end, this study investigated the association between accelerated or delayed age-related brain structural changes and cognition in early-stage BDI.

**Methods:**

First episode patients with BDI (*n* = 80) underwent cognitive assessment to yield demographically normed composite global and domain-specific scores in verbal memory, non-verbal memory, working memory, processing speed, attention, and executive functioning. Structural magnetic resonance imaging data were also collected from all participants and subjected to machine learning to compute the brain-predicted age difference (brainPAD), calculated by subtracting chronological age from age predicted by neuroimaging data (positive brainPAD values indicating age-related acceleration in brain structural changes and negative values indicating delay). Patients were divided into tertiles based on brainPAD values, and cognitive performance compared amongst tertiles with ANCOVA.

**Results:**

Patients in the lowest (delayed) tertile of brainPAD values (brainPAD range −17.9 to −6.5 years) had significantly lower global cognitive scores (*p* = 0.025) compared to patients in the age-congruent tertile (brainPAD range −5.3 to 2.4 yrs), and significantly lower verbal memory scores (*p* = 0.001) compared to the age-congruent and accelerated (brainPAD range 2.8 to 16.1 yrs) tertiles.

**Conclusion:**

These results provide evidence linking cognitive dysfunction in the early stage of BDI to apparent delay in typical age-related brain changes. Further studies are required to assess how age-related brain changes and cognitive functioning evolve over time.

## Introduction

Cognitive function in bipolar I disorder (BDI) shows evidence of heterogeneity. We and others have shown that patients with BDI can be stratified based on their cognitive functioning into groups displaying global cognitive impairment, selective cognitive impairment, and preserved or even above-average cognitive functioning (Bora et al., 2016; Burdick et al., 2014; Chakrabarty et al., 2020). Variability in cognitive function is also present during the premorbid period; population-based studies that have used scholastic performance as a proxy for cognitive function have found that the risk for BDI is higher at both the lowest and the highest levels of academic performance (Kendler, Ohlsson, Mezuk, Sundquist, & Sundquist, 2016), and that children with either low or high intelligence are at higher risk of developing BD (Papachristou et al., 2017; Parellada, Gomez-Vallejo, Burdeus, & Arango, 2017).

At present, there is no definitive model that can account for this heterogeneity in cognitive functioning. Initially, cognitive dysfunction in BDI was attributed to progressive changes occurring after illness onset, possibly as the cumulative result of medication exposure and neurotoxicity from increased systemic inflammation, oxidative stress and recurrent mood episodes (López-Jaramillo et al., 2010). Although there is evidence of cognitive decline around the time of disease onset (Lee et al., 2014; Zanelli et al., 2010), most longitudinal studies now suggest that progressive cognitive deterioration is not the rule in BDI, though it may occur in a subset of patients (Jiménez-López et al., 2019; Samamé, Cattaneo, Richaud, Strejilevich, & Aprahamian, 2021; Santos et al., 2014; Sánchez-Morla et al., 2019; Sparding et al., 2021; Torres et al., 2020). Deviation in maturational processes represents another plausible, but less studied, mechanism for cognitive dysfunction in BDI. Population-level data indicate that first episode of BD almost always occurs in adolescence/young adulthood, a critical period of neurodevelopment (Lewinsohn, Seeley, & Klein, 2003; Valli, Fabbri, & Young, 2019). Some studies have also shown that youth at clinical or familial high risk for BD show mild to moderate cognitive dysfunction, indicative of neurodevelopmental abnormalities (Bora, 2015). In our own sample of patients with first-episode mania (the same as used in the current study), those presenting with global cognitive dysfunction also had higher total gray matter volumes compared to healthy individuals (Chakrabarty et al., 2020), suggesting delay from typical maturational gray matter changes in adolescence/young adulthood (Dima et al., 2021; Frangou et al., 2021; Tamnes et al., 2017). It is therefore possible that the cognitive heterogeneity in BDI reflects different pathophysiological ‘pathways’, with neurodevelopmental and neuroprogressive mechanisms influencing the nature and/or degree of cognitive problems experienced by individual patients.

Structural brain imaging studies show significant age-related changes in brain morphometry throughout the lifespan that varies across individuals (Dima et al., 2021; Frangou et al., 2021; Wierenga et al., 2022). Application of machine algorithms to structural neuroimaging data enables an individual-level estimation of the biological age of the brain (brain-age) (Cole & Franke, 2017; Franke & Gaser, 2019). The difference between an individual's brain-age and their chronological age (referred to here as the brain-predicted age difference, or brainPAD) provides an indication as to whether age-related biological processes appear accelerated or delayed (Cole & Franke, 2017; Franke & Gaser, 2019). In the largest study to date on brainPAD in neuropsychiatric disorders, Kaufmann and colleagues reported that patients with chronic BDI had, on average, a moderate increase in brainPAD compared to healthy individuals (effect size *d* = 0.29), suggesting apparent acceleration in age-related brain structural changes in patients with established BD (Kaufmann et al., 2019). Conversely, a study in early-stage BD found that brainPAD was lower in patients compared to healthy individuals (*d* = −0.23) (Hajek et al., 2019). Collectively, there is some support for the notion that while a moderate degree of age-acceleration may be present in chronic cases, delayed brain maturation may be present in early-stage BD. During typical development, general intellectual ability and multiple aspects of executive function have been primarily associated with age-related reduction in cortical thickness, possibly reflective of synaptic pruning and improved synaptic efficiency during brain maturation, which continues well into the 2nd and 3rd decade of life (de Chastelaine, Donley, Kennedy, & Rugg, 2019; Porter, Collins, Muetzel, Lim, & Luciana, 2011; Schnack et al., 2015; Squeglia, Jacobus, Sorg, Jernigan, & Tapert, 2013). This and our previous findings suggest that a delay in normal neurodevelopmental trajectories may contribute to cognitive deficits early in the course of BD (Chakrabarty et al., 2020). In this context, the present study is the first to examine associations between brainPAD and cognitive functioning in a sample of patients with first-episode mania. Based on our previous data, we hypothesized that cognitive dysfunction across domains would be most pronounced in patients with a negative brainPAD, indicative of delayed maturation.

## Materials and methods

### Participants

Clinically stable patients meeting Diagnostic and Statistical Manual of Mental Disorders, fourth edition, text revision (DSM-IV TR) criteria for BDI were enrolled in the Systematic Treatment Optimization Program for Early Mania [STOP-EM] from UBC Hospital (UBCH) and affiliated sites, and community referrals (Association, 2000). The complete protocol has been previously described (Yatham, Kauer-Sant'Anna, Bond, Lam, & Torres, 2009). Patients in the STOP-EM program were recruited based on the following eligibility criteria: (1) age 14–35 years; (2) experienced the first manic/mixed episode in the preceding 3 months; (3) clinically stable and receiving mood-stabilizing treatment at enrollment; (4) no lifetime history of neurological disorder or traumatic brain injury; (5) sufficient English proficiency to understand and follow study procedures.

### Clinical procedures

Board-certified psychiatrists confirmed the diagnostic status (including substance and other comorbidities) of all participants via the Mini International Neuropsychiatric Interview (M.I.N.I.) (Sheehan et al., 1998). Mood symptoms were assessed with the Montgomery-Asberg Depression Rating Scale (MADRS) and the Young Mania Rating Scale (YMRS) (Montgomery & Asberg, 1979; Young, Biggs, Ziegler, & Meyer, 1978).

### Cognitive assessment

The cognitive assessment battery included tests which have previously been shown to be directly relevant to BD (Chakrabarty et al., 2020; Torres et al., 2014). All participants were administered the North American Adult Reading Test (NAART) to assess premorbid IQ (Bain, 2010; Uttl, 2002), while current cognitive function was evaluated using the Cambridge Neuropsychological Test Automated Battery (CANTAB) and additional tests from the Measurement and Treatment Research to Improve Cognition in Schizophrenia (MATRICS) Cognitive Consensus Battery (MCCB) (Nuechterlein et al., 2008). Details of individual tests and their corresponding domains are shown in online Supplementary Table S1. Test scores were transformed into demographically corrected z-scores based on normative data derived from healthy populations external to this study and provided in individual test manual (Delis, Kramer, Kaplan, & Ober, 2000; Golden, 2002; Lezak & Lezak, 2004; Reitan & Wolfson, 1993; Robbins et al., 1994; Wechsler, 1997). Demographically corrected test z-scores were averaged to obtain domain scores (Torres et al., 2014). Processing speed *z*-scores were coded such that negative scores indicated slower performance. Similar to the MATRICS composite score, a composite global cognition score was calculated as the average of the *z*-scores of the individual cognitive domains. Standardized NAART full-scale IQ scores were also calculated for each subject (Blair & Spreen, 1989).

### Imaging

Whole-brain T1-weighted magnetic resonance images were acquired on a Philips Achieva 3.0 Tesla scanner (Koninklijke Philips N.V, The Netherlands) using a three-dimensional axial inversion recovery-weighted spoiled gradient recalled sequence and the following parameters: FOV = 20 cm (RL) × 25.6 cm (AP), ACQ matrix = 256 × 256, isotropic image voxels (1 × 1 × 1 mm^3^), autoshim, TR/TE = autoset shortest, T/R head coil, flip angle = 8 degrees, and 1 mm thick contiguous 180 slices of the whole brain.

Cortical reconstruction and subcortical segmentation of the T1-weighted datasets were implemented in FreeSurfer image analysis suite (version 7.1.0; http://surfer.nmr.mgh.harvard.edu/). Processing included removal of non-brain tissue using a hybrid watershed/surface deformation procedure, segmentation of the subcortical white matter and deep gray matter volumetric structures, intensity normalization, tessellation of the boundary of gray matter and white matter, automated topology correction and surface deformation following intensity gradients to optimally place the gray/white and gray/cerebrospinal fluid borders at the location where the greatest shift in intensity defines the transition to the other tissue class. The Desikan-Killiany atlas was used to parcellate the brain into 68 cortical regions (Desikan et al., 2006), while subcortical segmentation was performed using the probabilistic atlas in FreeSurfer (Fischl et al., 2004). This procedure yielded measures of total intracranial volume (ICV), regional cortical thickness, surface area and subcortical volumes.

Participants with high-quality T1-images and high-quality FreeSurfeer parcellation and segmentation were included. Quality assessment of images involved two steps:

- Using visual inspection, each T1-weighted scan was assessed using 4 criteria: (1) image affected by movement, (2) temporal poles missing (even partly) in the reconstruction, (3) other parts of the cortex missing in the reconstruction, (4) non-brain tissue (e.g. dural/skull) still visible in the reconstructed pial surface. These criteria were applied separately for the left and right hemisphere. Each criterion was scored as 0 if there were ‘no errors visible’ or as 1 if there were ‘errors visible in at least 3 consecutive slices’. Scans with total scores of 1 or 2 were considered of good quality; those with higher scores were excluded.
- The quality of the FreeSurfer parcellation and segmentation of the T1-scans included after visual inspection was assessed using the Qoala-T tool (Klapwijk, van de Kamp, van der Meulen, Peters, & Wierenga, 2019) which uses a pre-trained supervised-learning model to classify each individual's FreeSurfer output as good or poor.

### Estimation of BrainPAD

We used a model developed by the Enhancing NeuroImaging Genetics through Meta-analysis (ENIGMA) Consortium to obtain brain-based age estimates (Han et al., 2020). The model was trained and validated using the same Freesurfer outputs as in the current study, which were derived from a sample of 4314 healthy individuals (43.3% males; age range: 18–75 years). Because the literature suggests sex differences in age-related brain structural changes (Jahanshad & Thompson, 2017), the ENIGMA model was specified separately for males and females. Accordingly, the parameters from the ENIGMA model were applied to the data from male and female participants of the current study to obtain brain-age estimates. Subsequently, the brainPAD for each participant was computed by subtracting chronological age from brain-age, and expressed in units of years. Positive brainPAD values indicate apparent acceleration in age-related brain structural changes. Conversely, negative values indicate apparent delay in age-related brain structural changes. In subsequent analyses, chronological age was included as a covariate to remove any residual effect on brainPAD (Le et al., 2018).

### Statistical analysis

As we were primarily interested in examining the degree of cognitive dysfunction in individuals at the extremes of the brainPAD distribution (i.e. showing the greatest degree of delay or acceleration compared to chronological age), the patient cohort was divided into tertiles based on brainPAD values, resulting in 3 groups: (1) a ‘delayed’ group (with brainPAD values in the lowest tertile); (2) an ‘age-congruent’ group (intermediate tertile, suggesting the least discrepancy between predicted brain-age and chronological age); and (3) an ‘accelerated’ group (with brainPAD values in the highest tertile). Mean values of demographically normed global cognition and individual domain z-scores were calculated for the delayed, age-congruent and accelerated brainPAD tertiles. The primary analysis was a comparison of global cognitive *z*-scores amongst tertiles, using ANCOVA correcting for chronological age, with a significance threshold of *p* ⩽ 0.05. To determine the specific nature of cognitive changes amongst tertiles, differences in individual domain *z*-scores were also assessed, with a Bonferroni correction for multiple comparisons (*p* = 0.05/6 individual domains = 0.008). Omnibus tests reaching the a priori level of significance were followed with Bonferroni corrected pairwise comparisons. To quantify the magnitude of differences amongst tertiles, Cohen's d effect sizes using the delayed tertile as a reference group were calculated.

Exploratory analyses compared differences in demographic or clinical variables (e.g. total illness duration, age of index episode, number of previous depressive episodes, psychotic features in mania, years of education, comorbid substance use, lithium/valproate/antipsychotic use) amongst tertiles, using ANCOVA controlling for chronological age or χ^2^ test, as appropriate. As these analyses were exploratory in nature, no correction for multiple comparisons was applied.

## Results

### Participant characteristics

Of the 91 participants who completed baseline cognitive assessment, 82 completed MRI scan at baseline. A further 2 of these were excluded due to poor scan quality, leaving a final sample of 80 participants with first-episode BDI. The demographic, clinical and cognitive characteristics of the entire patient group are shown in Tables 1 and 2.

**Table 1.** Demographic and clinical characteristics of entire first episode BDI patients and patients by brainPAD tertile

	BDI (*n* = 80)	Delayed brainPAD tertile (*n* = 27)	Age-congruent brainPAD tertile (*n* = 26)	Accelerated brainPAD tertile (*n* = 27)	Comparison of brainPAD tertiles
Female Sex *N* (%)	41 (51%)	12 (44%)	12 (46%)	17 (63%)	χ^2^ = 2.25 *p* = 0.324
Chronological Age [M (s.d.)]	22.50 (4.60)	22.37 (5.54)	24.38 (4.08)	20.81 (3.32)	*F*_(2,79)_ = 4.35 *p* = 0.016* age-congruent > accelerated (*p* = 0.004)
Years of Education [M (s.d.)]	13.60 (2.52)	12.96 (2.50)	14.81 (2.71)	13.07 (1.94)	*F*_(2,79)_ = 2.45 *p* = 0.093
Premorbid IQ [M (s.d.)]	105.81 (7.65)	105.16 (7.98)	107.42 (8.81)	104.90 (5.95)	*F*_(2,79)_ = 0.29 *p* = 0.751
YMRS [M (s.d.)]	1.61 (3.33)	2.89 (4.57)	0.96 (1.69)	0.96 (2.75)	*F*_(2,79)_ = 3.11 *p* = 0.050* delayed > age-congruent (*p* = 0.047), accelerated (*p* = 0.029)
MADRS [M (s.d.)]	5.90 (7.00)	7.62 (8.92)	6.00 (6.64)	4.15 (4.70)	*F*_(2,79)_ = 1.25 *p* = 0.292
Age at index episode^a^ [M (s.d.)]	19.65 (5.05)	19.27 (5.55)	21.85 (5.03)	17.76 (3.64)	*F*_(2,76)_ = 1.49 *p* = 0.232
Years of illness^b^[M (s.d.)]	2.80 (4.16)	3.32 (5.59)	2.27 (3.17)	2.84 (3.42)	*F*_(2,76)_ = 1.20 *p* = 0.306
Number of previous depressive episodes [M (s.d.)]	1.32 (2.28)	1.04 (1.64)	1.15 (1.67)	1.77 (3.19)	*F*_(2,76)_ = 1.73 *p* = 0.184
Psychosis in first episode mania, *N* (%)	57 (71%)	20 (77%)	22 (85%)	15 (56%)	χ^2^ = 6.01 *p* = 0.050* age-congruent > accelerated (*p* = 0.018)
On lithium, *N* (%)	38 (47%)	17 (63%)	10 (38%)	11 (41%)	χ^2^ = 3.93 *p* = 0.140
On valproate, *N* (%)	31 (39%)	8 (30%)	12 (46%)	11 (41%)	χ^2^ = 1.59 *p* = 0.451
On antipsychotic *n* (%)	60 (75%)	18 (67%)	19 (73%)	23 (85%)	χ^2^ = 2.54 *p* = 0.280
Lifetime drug or alcohol abuse *n* (%)	28 (35%)	11 (39%)	10 (36%)	7 (25%)	χ^2^ = 1.76 *p* = 0.415

YMRS, Young Mania Rating Scale; MADRS, Montgomery Asberg Depression Rating Scale.

a Age at first mood episode, either depressive, hypomanic or manic.

b Years between index episode (either depressive, hypomanic or manic) and assessment.

*p ⩽ 0.05.

**Table 2.** Comparison of demographically normed cognitive z-scores amongst brainPAD tertiles

	All BDI participants (*n* = 80)	Delayed brainPAD tertile (*n* = 27)	Age-congruent brainPAD tertile (*n* = 26)	Accelerated brainPAD tertile (*n* = 27)	Comparison brainPAD tertiles	Effect size age-congruent v. delayed (Cohen's d)	Effect size accelerated v. delayed (Cohen's d)
Global cognition [M (s.d.)]	−0.15 (0.56)	−0.39 (0.62)	0.02 (0.47)	−0.08 (0.51)	*F*_(2,76)_ = 3.90 *p* = 0.025* age-congruent > delayed (*p* = 0.031)	0.74	0.53
Verbal memory [M (s.d.)]	−0.15 (1.06)	−0.76 (1.16)	0.22 (0.91)	0.09 (0.84)	*F*_(2,79)_ = 7.76 *p* = 0.001* age-congruent > delayed (*p* = 0.005) accelerated > delayed (*p* = 0.003)	0.94	0.84
Working memory [M (s.d.)]	−0.14 (0.89)	−0.42 (1.03)	0.05 (0.78)	−0.05 (0.79)	*F*_(2,79)_ = 1.98 *p* = 0.146	0.51	0.40
Executive functioning [M (s.d.)]	−0.04 (0.70)	−0.24 (0.77)	−0.02 (0.60)	0.13 (0.68)	*F*_(2,79)_ = 2.18 *p* = 0.120	0.33	0.51
Nonverbal memory [M (s.d.)]	0.07 (0.74)	−0.13 (0.87)	0.11 (0.57)	0.24 (0.74)	*F*_(2,78)_ = 1.89 p = 0.158	0.32	0.45
Attention [M (s.d.)]	−0.13 (0.90)	−0.20 (0.88)	0.17 (0.84)	−0.37 (0.92)	*F*_(2,76)_ = 2.43 *p* = 0.095	0.44	−0.19
Processing speed^a^ [M (s.d.)]	−0.46 (0.70)	−0.57 (0.60)	−0.42 (0.80)	−0.39 (0.71)	*F*_(2,79)_ = 0.49 *p* = 0.615	0.21	0.27

a Negative processing speed scores indicate slower performance.

*Significant at level of *p* ⩽ 0.05 for global cognition or *p* ⩽ 0.008 for individual cognitive domains.

### Participant brainPAD and clinical/demographic variables

Mean brainPAD of patients was −1.00 (s.d. 8.22), indicating a small delay in normative age-related brain structural changes in the whole sample. Patients in the lowest (delayed) tertile (*n* = 27) had a mean brainPAD of −10.79 (s.d. 2.99), with brainPAD values ranging −17.95 to −6.49. Those in the age-congruent tertile (*n* = 26) had mean brainPAD of −1.45 (s.d. 2.49; range −5.30 to 2.38) and those in the accelerated tertile (*n* = 27) had a mean brainPAD of 7.79 (s.d. 3.73; range 2.81 to 16.10). The distribution of brainPAD values is shown in online Supplementary Fig. S1.

The demographic and clinical characteristics of patients in each brainPAD tertile are shown in Table 1. The accelerated tertile had a lower mean chronological age (*p* = 0.004) and a lower proportion of individuals with psychotic features in mania (*p* = 0.018) compared to the age-congruent tertile. The delayed tertile had higher YMRS scores compared to age-congruent (*p* = 0.047) and accelerated (*p* = 0.029) tertiles. There were no differences amongst brainPAD tertiles in any other clinical or demographic variable, including premorbid IQ (Table 1).

### Cognitive performance in patients split by brainPAD tertiles

**Fig. 1.**
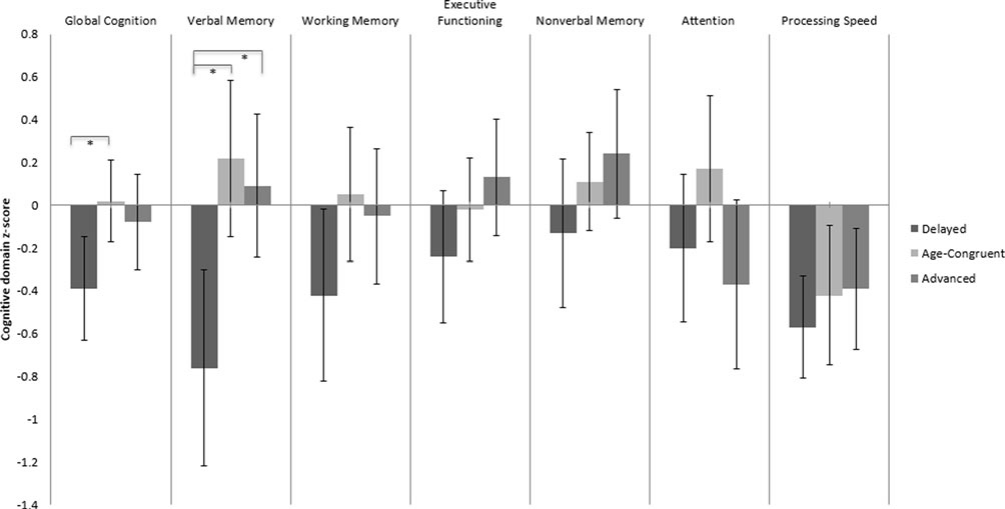
Cognitive performance in delayed, age-congruent and accelerated brainPAD tertiles. Mean demographically normed cognitive domain *z*-scores with 95% CI shown. *Significant difference following correction for multiple comparisons.

Normative cognitive scores for each tertile are shown in Table 2 and Fig. 1. In ANCOVA analyses controlling for age, there were significant differences in global cognitive scores amongst tertiles (*F*_(2,76)_ = 3.90, *p* = 0.025), with post-hoc testing showing the delayed tertile scoring significantly lower than the age-congruent tertile (*p* = 0.031), with a moderate effect size (*d* = 0.74). Verbal memory also significantly differed amongst tertiles (*F*_(2,79)_ = 7.76, *p* = 0.001), with the delayed tertile scoring significantly lower than the age-congruent (*p* = 0.005, *d* = 0.94) and accelerated (*p* = 0.003, *d* = 0.84) tertiles, with large effect sizes. There were no differences between accelerated and age-congruent tertiles in post-hoc analyses. A plot of global cognition *v.* brainPAD values as a continuous measure is shown in online Supplementary Fig. S2.

ANCOVAs which additionally included YMRS scores or psychotic features in mania showed a similar pattern of significant results for global cognition and verbal memory (online Supplementary Table S2). After controlling for psychosis, tertiles showed trend-level differences in attention scores, with the accelerated tertile scoring lower than age-congruent. This however did not survive correction for multiple comparisons (uncorrected *p* = 0.048).

## Discussion

Using a brain-age estimation model, we examined how deviations from typical age-related structural changes were associated with cognitive functioning in a sample of patients with BDI presenting with first-episode mania. Individuals in the delayed tertile of brainPAD values showed the greatest magnitude of global cognitive impairments, with moderate decrements compared to age-congruent patients. Those in the delayed tertile also showed significant, large decrements in verbal memory compared to other patients.

These findings suggest that global cognitive impairment, and specifically verbal memory deficits, in the early stages of BD are related to an apparent delay in typical age-related brain changes. This is aligned with prior observations that in a subset of patients, BD is associated with abnormalities in neurodevelopment characterized by early childhood subthreshold manic-like symptoms and suboptimal premorbid cognitive function (Papachristou et al., 2017; Seidman et al., 2013). In the Tracking Adolescents' Individual Lives Survey (TRAILS), a prospective Dutch community cohort (*n* = 2230), children with high manic-like symptoms and low IQ had the highest risk of developing BDI at age 19, although most TRAILS participants presenting with BDI had no cognitive difficulties as children (Papachristou et al., 2017). Other studies have similarly found that some patients with BD display compromised childhood/adolescent intellectual and adaptive functioning, indicative of neurodevelopmental difficulties in a subset of patients (MacCabe et al., 2010; Parellada et al., 2017). Our results are also in line with previous analyses of this first episode cohort showing that patients with global cognitive and verbal memory deficits displayed gray matter volumetric changes suggestive of a delay in normal adolescent brain maturational processes (Chakrabarty et al., 2020; Chakrabarty, Kozicky, Torres, Lam, & Yatham, 2015). This study, which demonstrates that first-episode patients with the highest degree of delay from normal neurodevelopmental trajectories show the most pronounced cognitive deficits, lends support to the hypothesis that a subset of patients with BDI experience an aberrant neurodevelopmental course while also clarifying the nature of these neurodevelopmental changes.

Notably, patients in the delayed brainPAD tertile showed the most pronounced deficits in verbal memory, scoring close to 1 standard deviation below the normative mean and significantly lower than other patients in this cohort. Verbal memory is a core deficit in BDI, with multiple studies finding dysfunction in first-episode patients and in euthymia (Chakrabarty et al., 2015), and has also been consistently associated with social and occupational functioning in BD (Bonnín Cdel et al., 2014; Lomastro, Valerio, Blasco, Tagni, & Martino, 2020; Martinez-Aran et al., 2007; Szmulewicz et al., 2018; Torres et al., 2011). Our results provide a potential neurobiological substrate for this deficit. Conversely, all brainPAD tertiles showed some degree of processing speed deficits, with normative scores ranging from −0.57 to −0.39. Some, (Daban et al., 2012; Glahn et al., 2010) but not all (Luperdi et al., 2019), studies have suggested that delays in processing speed may be a marker of genetic vulnerability to BD; our results similarly indicate that processing speed deficits may be a trait feature in early BD. Interestingly, brainPAD was not associated with premorbid IQ. This may be due to the measure used in this study to assess premorbid IQ. While the NAART provides a reliable estimate of premorbid crystallized intelligence, it has limited utility in predicting premorbid abilities in cognitive domains such as memory, processing speed and executive functioning (Frick, Wahlin, Pachana, & Byrne, 2011; Schretlen, Buffington, Meyer, & Pearlson, 2005). The NAART therefore may not capture dysfunction in these domains during the neurodevelopmental period. The NAART is also heavily influenced by education; the limited variability in years of education amongst tertiles in this cohort may have restricted ability to detect differences in the NAART (Hayat et al., 2016; Uttl, 2002).

Individuals in the accelerated tertile did not show statistically significant differences compared to the age-congruent tertile in any cognitive measure. One possibility is that the sample was underpowered to detect significant differences in this tertile. The accelerated tertile displayed the lowest attention scores which, after controlling for the presence of psychotic features in mania, were lower than the age-congruent tertile at an uncorrected threshold of significance. In a larger sample, this difference may have reached the corrected level of significance. Another possible explanation for this negative finding is the first episode cohort used in this analysis. Some, though not all (Serafini et al., 2021), individuals with BD longitudinally display cognitive decline and progressive structural brain changes, both of which have been linked to number of mood episodes (Abé et al., 2015; Chen et al., 2021; Sánchez-Morla et al., 2019). Consistent with this idea of a subgroup of patients displaying evidence of neuroprogression with time and mood recurrence, those with accelerated brain aging may show more pronounced cognitive deficits in a cohort with longer duration of illness.

This study has a number of strengths, foremost of which is the inclusion of a well characterized, exclusively first episode mania cohort. It also represents the first examination of brain-age and cognition in BD; to our knowledge, there has only been one previous study of brain-age and cognition in mood disorders, which examined middle age and geriatric MDD (Christman et al., 2020). However, some limitations and unanswered questions remain. The cross-sectional nature of the dataset precludes any causative associations and does not inform either about the timing of the potential developmental delay nor about the evolution of age-related brain changes following illness onset. To answer this, a well powered longitudinal study assessing cognition and brain structure over multiple timepoints is needed. The longitudinal impact of mood stabilizer and comorbid conditions requires further examination. Additionally, higher brain-age relative to chronological age (i.e. higher brainPAD) has been associated with adverse physical outcomes and cognitive decline in mid-life and older adults (Anatürk et al., 2021; Cole et al., 2018; Elliott et al., 2019; Ronan et al., 2016). It would therefore be of interest to examine longitudinal associations between brainPAD and physical health markers, and associations between brainPAD, physical health and cognitive functioning in older adults with BD. Patient participants in this cohort were generally high functioning and had preserved premorbid intelligence, potentially limiting generalizability of these results to other patient cohorts. Information regarding family history of neurodevelopmental disorders and birth trauma was not systematically collected in this cohort, but may be additional variables of interest associated with cognitive functioning and predicted brain-age. However, this study provides a useful starting point for understanding the potential neurodevelopmental basis of cognitive deficits in BD.

## Supplementary material

For supplementary material accompanying this paper visit https://doi.org/10.1017/S0033291722002136. click here to view supplementary material
