# Supplementary material for: Brain age and cognitive functioning in first-episode bipolar disorder
Source: Psychol Med. 2022 Jul 25;53(11):5127–35. doi: 10.1017/S0033291722002136 (PMC10476063; doi:10.1017/S0033291722002136)
Supplement: Supplementary file 1 [file S0033291722002136sup001.zip › S0033291722002136sup001.docx]

**Supplementary Table 1: Tests used to generate cognitive domain scores**

| **Cognitive Domain** | **Included Individual Test Scores** |
| --- | --- |
| Processing speed | Trail Making Test Part A  Stroop Color: trials number correct  Stroop Word: trials number correct  Letter Fluency: number correct |
| Attention | CANTAB Rapid Visual Information Processing: discriminability score  CANTAB Rapid Visual Information Processing: latency score |
| Verbal Memory | California Verbal Learning Test 2^nd^ edition (CVLT-II): total recall trials 1-5  CVLT-II: long delay free recall |
| Nonverbal Memory | CANTAB Pattern Recognition Memory: number correct  CANTAB Spatial Recognition Memory: number correct  CANTAB Paired Associate Learning: total number of errors |
| Working Memory | Letter/Number Sequencing  CANTAB Spatial Working Memory: between errors |
| Executive Functioning | Trail Making Test Part B  Stroop Color-Word: trials number correct  CANTAB Intra-Extra Dimensional set shifting task: number of extra-dimensional shifting errors  CANTAB Stockings of Cambridge: number of problems solved |
