# Supplementary material for: Brain age and cognitive functioning in first-episode bipolar disorder
Source: Psychol Med. 2022 Jul 25;53(11):5127–35. doi: 10.1017/S0033291722002136 (PMC10476063; doi:10.1017/S0033291722002136)
Supplement: Supplementary file 1 [file S0033291722002136sup001.zip › S0033291722002136sup002.docx]

**Supplemental Table 2:** Comparison of demographically normed cognitive z-scores amongst brainPAD tertiles, controlling for manic symptom severity and psychotic features in mania

|  | **All BDI participants (n=80)** | **Delayed brainPAD tertile (n=27)** | **Age-congruent brainPAD tertile (n=26)** | **Accelerated brainPAD tertile (n=27)** | **Comparison brainPAD tertiles (covariates age and YMRS)** | **Comparison brainPAD tertiles (covariates age and psychosis in FEM)** |
| --- | --- | --- | --- | --- | --- | --- |
| Global cognition [M (SD)] | -0.15  (0.56) | -0.39  (0.62) | 0.02  (0.47) | -0.08 (0.51) | F(2,76)=3.33  p=0.042*  age-congruent>  delayed (p=0.049) | F(2,75)=3.64 p=0.031*  age-congruent>  delayed (p=0.031) |
| Verbal memory [M (SD)] | -0.15  (1.06) | -0.76  (1.16) | 0.22  (0.91) | 0.09 (0.84) | F(2,79)=6.82  p=0.002*  delayed<age-congruent (p=0.008),  accelerated  (p=0.005) | F(2,78)=7.33 p=0.001*  delayed<age-congruent (p=0.004),  accelerated  (p=0.007) |
| Working memory [M (SD)] | -0.14  (0.89) | -0.42 (1.03) | 0.05  (0.78) | -0.05 (0.79) | F(2,79)= 1.52  p=0.225 | F(2,78)=1.70 p=0.190 |
| Executive functioning [M (SD)] | -0.04  (0.70) | -0.24 (0.77) | -0.02 (0.60) | 0.13 (0.68) | F(2,79)= 1.82  p=0.169 | F(2,78)=1.87 p=0.162 |
| Nonverbal memory [M (SD)] | 0.07  (0.74) | -0.13 (0.87) | 0.11  (0.57) | 0.24 (0.74) | F(2,78)=2.04  p=0.137 | F(2,77)=1.60 p=0.210 |
| Attention [M (SD)] | -0.13  (0.90) | -0.20 (0.88) | 0.17  (0.84) | -0.37 (0.92) | F(2,76)=2.36  p=0.102 | F(2,75)=3.17  p=0.048 |
| Processing speed^t^ [M (SD)] | -0.46  (0.70) | -0.57 (0.60) | -0.42 (0.80) | -0.39 (0.71) | F(2,79)=0.53  p=0.592 | F(2,78)=0.73  p=0.486 |

FEM, first episode mania; YMRS, Young Mania Rating Scale

^t^Lower processing speed scores indicate slower performance

*Significant at level of p<0.05 for global cognition or p<0.008 for individual cognitive domains
