# Supplementary figures and images for: Brain age and cognitive functioning in first-episode bipolar disorder
Source: Psychol Med. 2022 Jul 25;53(11):5127–35. doi: 10.1017/S0033291722002136 (PMC10476063; doi:10.1017/S0033291722002136)

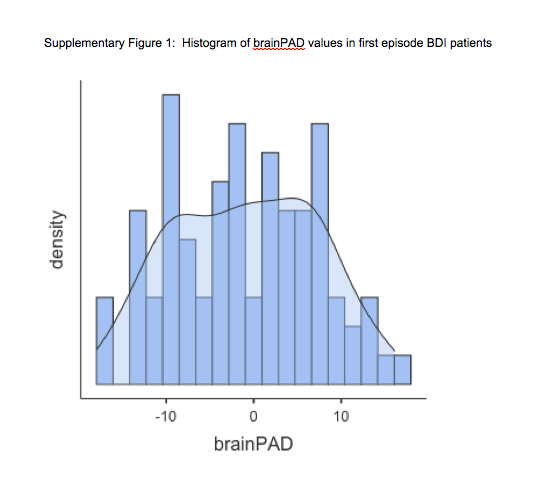

Supplement: Supplementary file 1 [file S0033291722002136sup001.zip › S0033291722002136sup004.tiff]

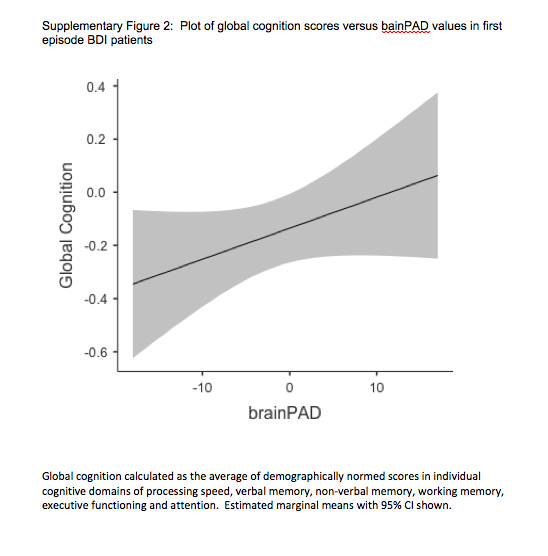

Supplement: Supplementary file 1 [file S0033291722002136sup001.zip › S0033291722002136sup006.tiff]
